# Precipitation and plant cover promote soil organic carbon accumulation in the northeastern Qinghai-Tibet Plateau

**DOI:** 10.1093/aobpla/plag024

**Published:** 2026-06-02

**Authors:** Xin Wang, Yanbin Wen, Huiyu Song, Na Wang, Huakun Zhou, Guiling Wu, Yangong Du

**Affiliations:** Northwest Institute of Plateau Biology, Chinese Academy of Sciences, Xining, Qinghai Province 810008, China; University of Chinese Academy of Sciences, Beijing 100049, China; Northwest Institute of Plateau Biology, Chinese Academy of Sciences, Xining, Qinghai Province 810008, China; University of Chinese Academy of Sciences, Beijing 100049, China; Northwest Institute of Plateau Biology, Chinese Academy of Sciences, Xining, Qinghai Province 810008, China; University of Chinese Academy of Sciences, Beijing 100049, China; Northwest Institute of Plateau Biology, Chinese Academy of Sciences, Xining, Qinghai Province 810008, China; University of Chinese Academy of Sciences, Beijing 100049, China; Northwest Institute of Plateau Biology, Chinese Academy of Sciences, Xining, Qinghai Province 810008, China; University of Chinese Academy of Sciences, Beijing 100049, China; Qinghai University, Xining, Qinghai Province 810016, China; Northwest Institute of Plateau Biology, Chinese Academy of Sciences, Xining, Qinghai Province 810008, China; University of Chinese Academy of Sciences, Beijing 100049, China

**Keywords:** mean annual precipitation, plant cover, plant diversity, soil organic carbon accumulation

## Abstract

As a core region for global high-altitude carbon cycling, the Qinghai-Tibet Plateau is a key region influencing the regulation of the global terrestrial carbon balance. However, the mechanisms controlling the accumulation of soil organic carbon (SOC) in this fragile ecosystem remain unclear. We surveyed 98 sample sites in the northeastern Qinghai-Tibet Plateau and analysed the data using Bayesian statistical methods. The 0–20 cm SOC storage in the Qinghai region is ∼0.37 Pg. Horizontally, SOC exhibited a decreasing trend from east to west, while vertically, it decreased with increasing soil depth, with a reduction of 24.28%. The result showed that plant cover and mean annual precipitation (MAP) were the dominant positive drivers of SOC accumulation in both soil layers (the 95% credible interval did not overlap with zero). Notably, plant cover and mean annual temperature had stronger effects on SOC in the surface layer. The Bayesian structural equation model indicated that plant cover, MAP, Shannon diversity index, and the litter *C/N* ratio collectively explained 68% of the variation in SOC across both layers. The path coefficient (standardized effect size) of MAP on SOC increased with soil depth, whereas that of plant cover decreased, indicating that the influence of environmental factors on SOC accumulation is dependent on depth. These results demonstrate that accurately predicting the Qinghai-Tibet Plateau’s SOC accumulation necessitates the explicit consideration of local plant cover conditions and concurrent precipitation shifts regarding existing SOC accumulation.

## Teaser

Using Bayesian models on 98 sampling sites across the northeastern Qinghai–Tibet Plateau, we found that soil organic carbon (SOC) decreases from east to west and is significantly higher in surface than in subsurface soils. Plant cover and mean annual precipitation are the key drivers of SOC accumulation, and the influence of these environmental factors on SOC is depth dependent.

## Introduction

The soil organic carbon (SOC) pool in the top metre of soil is approximately three-fold higher than the living vegetation carbon pool and twice that of the atmospheric carbon pool (Lal 2004). In China, SOC storage in the top 1 m of terrestrial ecosystems (including forests, grasslands, wetlands, croplands, and deserts) ranges from 82 to 89 Pg, with 38%–42% stored in the top 0–20 cm layer (Wu et al. 2024). On the Qinghai-Tibet Plateau, permafrost regions store ∼17 Pg of SOC in the upper 2 m, and this stock is projected to decline under future climate scenarios (Zhao et al. 2018). In the Qinghai Lake Basin, SOC in the 0–30 cm layer accounts for ∼43% of the total profile storage, highlighting that a large proportion of organic carbon is held in surface soils across the plateau (Ma et al. 2022). The Qinghai-Tibet Plateau, often referred to as the ‘Roof of the World’ owing to its high-elevation, low-temperature environment, acts as a crucial climate change indicator in the Northern Hemisphere (Qiu 2008) and harbours significant yet vulnerable SOC stocks (Wang et al. 2026). The SOC accumulation process governs terrestrial ecosystem carbon storage and global carbon budgets (Ajami et al. 2016) and regulates the ecosystem carbon balance, ultimately determining whether soils function as net carbon sources or sinks on regional and global scales (Román Dobarco et al. 2023). Consequently, understanding the drivers of SOC accumulation on the Qinghai-Tibet Plateau is critical for advancing carbon management strategies and achieving carbon neutrality targets.

Climate conditions, particularly temperature and precipitation, are recognized as dominant environmental factors influencing SOC by regulating both carbon input and SOC decomposition processes. Warming may increase plant productivity and facilitate carbon input (Wang et al. 2025a). However, climate warming may enhance microbial decomposition, thereby stimulating soil carbon release (Crowther et al. 2016). A global-scale study indicated that increased precipitation can offset SOC losses induced by climate warming, with this compensatory effect persisting across soil depths but varying significantly among ecosystems (Wang et al. 2025b). Precipitation, particularly in water-limited ecosystems, indirectly enhances soil carbon input primarily by promoting vegetation growth and increasing net primary productivity (Weltzin et al. 2003, Wang et al. 2025a), as well as by alleviating water stress, thereby reducing the decline in vegetation productivity caused by water deficit (Felton et al. 2021). However, another research that combined warming and reduced precipitation decreased soil moisture more significantly than either warming or reduced precipitation alone (Liu et al. 2009, Wei et al. 2023). Reduced soil moisture inhibits plant growth and physiological processes (Ciais et al. 2005), thereby leading to a significant decline in SOC (Wei et al. 2023). In summary, temperature and precipitation have complex and contrasting effects on SOC dynamics. Although both influence SOC through plant inputs and microbial decomposition, their relative importance in driving SOC accumulation on the Qinghai-Tibet Plateau remains unclear.

In addition to climatic factors, plants constitute the principal source of soil organic matter. Numerous studies have demonstrated that plant diversity typically enhances organic carbon levels via complementary effects, including resource distribution, niche partitioning, and biotic or abiotic interactions among species (Fornara and Tilman 2008, Lange et al. 2015). According to the niche complementarity hypothesis, community carbon storage capacity is primarily driven by coexisting species with distinct functional characteristics (Tilman et al. 1997, Mensah et al. 2016), implying that community species diversity is a key vegetation attribute determining SOC accumulation. Although this hypothesis has been widely validated in China’s subtropical ecosystems (Huang et al. 2025) and across cold temperate, temperate, subtropical, and tropical zones (Chen et al. 2018), its applicability in high-altitude, cold environments such as the Tibetan Plateau remains uncertain. Given the extreme low temperatures and short growing season on the Plateau, plant–plant interactions may shift from competition to facilitation (Callaway et al. 2002), potentially altering the relationship between plant diversity and SOC accumulation. Therefore, understanding how plant diversity influences SOC accumulation under such harsh conditions is essential for predicting soil carbon dynamics in this climate-sensitive region.

The influence of climatic and vegetative environmental factors on SOC accumulation may not be uniform across the soil profile. A study of Chinese forest ecosystems assessed the environmental controls on SOC accumulation across vertically stratified layers (0–20 vs. 20–100 cm), revealing that the effects of precipitation and the Shannon index on topsoil organic carbon accumulation were stronger than those in deeper soil layers (Zhou et al. 2019). The Plateau's cold climate suppresses microbial activity and slows the vertical transport and decomposition of organic matter, and this reduced turnover rate may decouple deep layer SOC dynamics from surface environmental conditions (Wang and Hu 2024). Therefore, how environmental factors control SOC accumulation across soil layers, and whether a consistent depth-dependent pattern exists, remains a critical knowledge gap on the Qinghai-Tibetan Plateau.

Previous studies investigating the environmental controls on SOC accumulation predominantly employed frequentist approaches. These methods treat regression coefficients as fixed constants, yielding single-value point estimates (e.g. via maximum likelihood) that fail to characterize the complete uncertainty profile (e.g. credible intervals or skewness of *β*). Their reliance on restrictive assumptions (e.g. linear relationships and normally distributed errors) may further introduce estimation bias when such assumptions are violated (Permai and Tanty 2018). In contrast, Bayesian analysis integrates domain knowledge with empirical data through the prior distribution and the likelihood of the posterior distribution (Permai and Tanty 2018). This approach generates posterior probability distributions rather than point estimates, enabling direct uncertainty quantification and effectively addressing the limitations of frequentist methods. Given these advantages, we implemented a Bayesian analysis at 98 sampling sites in the northeastern Qinghai-Tibet Plateau. These sites comprehensively represent almost all major regional vegetation types, ensuring ecological representation. Soils were stratified into two depths (surface: 0–10 cm; subsurface: 10–20 cm) during collection. We hypothesized that (i) precipitation is the dominant climatic driver of SOC accumulation because increased rainfall stimulates plant growth and directly enhances carbon input; (ii) plant diversity is a key vegetation attribute governing SOC accumulation, as species with distinct functions jointly drive carbon storage; and (iii) SOC accumulation responses to environmental factors show depth-dependent patterns in the northeastern Qinghai-Tibet Plateau because organic carbon sources, microbial activity, and decomposition conditions differ across soil layers.

## Materials and methods

### Study sites

This research was carried out in Qinghai Province, situated in the northeastern region of China’s Qinghai-Tibet Plateau (92°17′ E–101°44′ E and 32°18′ N–38°35′ N). Covering a total area of ∼72.23 × 10^4^ km^2^, Qinghai serves as the source region of the Yangtze, Yellow, and Lancang Rivers, earning it the designation ‘China’s Water Tower’. The province features highly heterogeneous topography, with elevations generally decreasing from west to east. The landscape comprises a mosaic of mountains, plateaus, basins, and river valleys, with altitudes ranging from 2980 to 4674 m. The area exhibits a typical continental climate characteristic of high-altitude plateaus, marked by long sunshine duration, strong solar radiation, high rates of evaporation and transpiration, and a high diurnal temperature range coupled with relatively stable yearly temperatures, with mean annual temperature (MAT) ranging from −7.38 to 3.46°C. Precipitation is scarce and unevenly distributed, with mean annual precipitation (MAP) varying from 209.80 to 792.40 mm in the region. The vegetation is diverse, comprising forests, shrublands, and meadows, with grasslands as the dominant land cover type. Major grassland types include temperate steppe, alpine meadow, lowland meadow, temperate meadow steppe, mountain meadow, herbaceous marsh, alpine steppe, and temperate desert steppe. The primary soil types in the region are meadow, alpine steppe, calcic (lime rich), and marsh soils, which reflect the region’s complex terrain and diverse ecological conditions.

### Field sampling and sample testing

#### Collection and processing of plant samples

**Figure 1:**
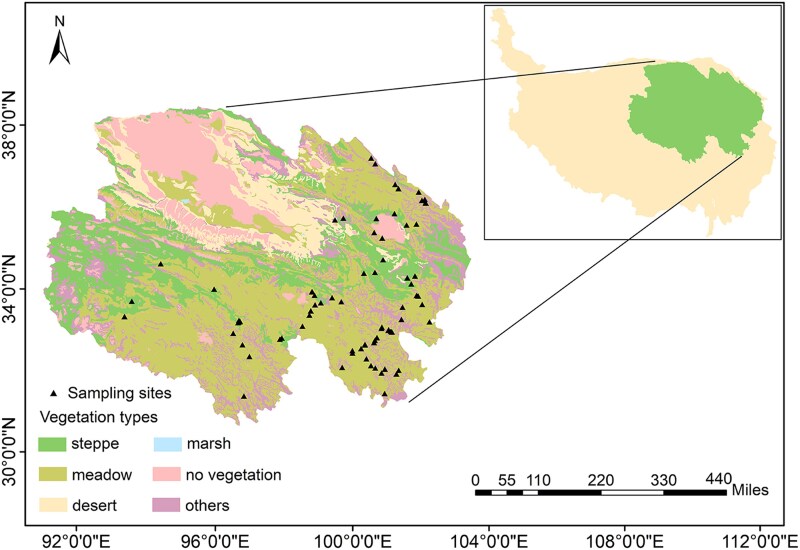
Spatial distribution of sampling sites on the northeastern Qinghai-Tibetan Plateau. Figure source: National Catalogue For Geographic Information (https://cloudcenter.tianditu.gov.cn/dataSource)

We selected 98 sample sites covering the main grassland types in Qinghai Province during the growing seasons (July to September) of 2011 and 2012 (Fig. 1 and Supplementary Table S1). Sampling locations were determined by comprehensively considering vegetation type, elevation, and accessibility. Within each sample site (100 m × 10 m), five (1 m × 1 m) quadrats were selected at 20 m intervals. After investigating the vegetation attributes within each quadrat, soil samples were collected. Plant height and cover were recorded within each quadrat. For each species present in the quadrat, five individual plants or clumps were randomly selected to measure the average height. The cover of each species within the sample quadrats was estimated by averaging the individual cover values measured by multiple (four) observers, which were then used to calculate species diversity. Above-ground biomass was collected by clipping method at each quadrat. The samples were then oven dried at 65°C to a constant weight. The oven-dried weight of the above-ground portions was regarded as the above-ground biomass. Litter samples were gathered from the soil surface. Subsequently, the collected above-ground plant litter was dried, finely ground, and used to determine its carbon and nitrogen concentrations.

#### Soil sample collection and preparation

Additionally, soil samples from the surface (0–10 cm) and subsurface (10–20 cm) layers were taken from each plot, where vegetation surveys were conducted, using a 5-point sampling method. A soil core was obtained using a 7-cm diameter auger, and samples from the same depth within each plot were combined to form a composite sample for that plot. The collected soil samples were transported to the laboratory, air-dried at room temperature, passed through a 2-mm sieve, and prepared for the determination of SOC content. Soil bulk density was assessed with the core-ring technique by measuring oven-dried soil mass within a known volume. In this study, litter and SOC were determined using the potassium dichromate external heating method (Guo et al. 2015), while the litter nitrogen was measured via the Kjeldahl method (Jaroonchon et al. 2010).

#### Acquisition of the climate and land cover data

MAT and MAP data were acquired from the China National Science and Technology Infrastructure Platform-National Earth System Science Data Center (http://www.geodata.cn). Specifically, we downloaded datasets for MAT (1982–2022) and MAP (1982–2022), both with a spatial resolution of 1 km. We acquired gridded climate datasets covering the period 2011–22. Using spatial analysis tools in ArcMap10.8, we extracted the MAP and MAT for each sampling site based on its geographic coordinates (latitude and longitude) and the corresponding sampling year. The land cover data for calculating SOC storage in Qinghai Province were obtained from the MODIS land cover product MCD12Q1 (Collection 6.1) on the Google Earth Engine (GEE) platform. We extracted the IGBP land cover band (LC_Type1) within the provincial boundary and applied GEE’s pixelArea () function to calculate pixel areas. Land cover types were then grouped to derive area statistics. The grassland area was identified based on the IGBP Grasslands class and summed to quantify its total extent across Qinghai Province. The average SOC stock was calculated across 98 sampling sites to represent the mean grassland SOC density (Mg C/ha) in Qinghai Province. Finally, the regional SOC storage was estimated by multiplying this average value by the total grassland area (459 367 km^2^), with results expressed in megagrams of carbon (Mg C) and further converted to petagrams of carbon (Pg C).

The calculation method for SOC storage was calculated as follows:


$$\mathrm{SO}C_{d}=\frac{\sum C_{i}\times H\times B}{100}$$

$$\mathrm{SO}C_{20}=\overset{n}{\underset{i=1}{\sum }}\;\mathrm{SO}C_{d,\;i}\times A$$

Here, $\mathrm{SO}C_{d}$ (kg/m^2^) represents the SOC density; $\mathrm{SO}C_{20}$ represents the 0–20 cm SOC storage; $C_{i},\;H,\;B$, $\mathrm{SO}C_{d,i}\;$, and *A* denote SOC content (g/kg), soil depth (cm), soil bulk density (g/cm^3^), the carbon density of the *i*th soil layer, and grasslands area (459 367 km^2^), respectively.

The calculation method for the Shannon–Wiener diversity index for species is as follows:


$$P_{i}=(h+c)/2$$

$$\mathrm{Shannon}\text{–}\mathrm{Wiener}\;\mathrm{index}=-\overset{s}{\underset{i=1}{\sum }}\;P_{i}\ln\;P_{i}$$

Here, $P_{i}$ represents the importance value of the *i*th species, and *h* and *c* denote the species’ relative height and relative cover, respectively. *S* indicates the total number of species in the sample plot.

The calculation method for litter *C/N* is as follows:


LCN=LC/LN

Here, *LCN* represents the litter *C/N*, *LC* refers to the litter carbon content, and *LN* refers to the litter nitrogen content.

#### Data analysis

This study employed a one-way analysis of variance to examine the spatial variability of SOC at depths. To elucidate the influence mechanisms of environmental factors on SOC accumulation, we first incorporated all environmental variables, including Shannon–Weiner diversity index, plant cover, MAT, MAP, litter *C*/*N* ratio, and above-ground biomass, into a Bayesian mixed-effects model. Then, we constructed a Bayesian structural equation model (BSEM) using MAP, plant cover, the Shannon–Wiener diversity index, and the litter *C*/*N* ratio. The Shannon–Wiener diversity index (Fayiah et al. 2019, Spohn et al. 2023) and litter *C*/*N* (Zhou et al. 2019) were included in the model due to their significant influence on SOC. Before conducting the data analysis, we performed collinearity and normality tests. First, logarithmic transformation was applied to MAP, Shannon–Wiener diversity index, litter *C*/*N* ratio, above-ground biomass, and plant cover to reduce data skewness. Subsequently, all variables (including the logarithmically transformed ones) were standardized to ensure the comparability of regression coefficients, thereby improving the reliability of the model results. In the Bayesian mixed-effects model, all six log-transformed and standardized environmental factors were included as fixed effects, with latitude and longitude as spatial autocorrelation correction terms using a squared exponential kernel function, and sites were treated as random effects. We used Moran’s I to examine the spatial autocorrelation of model residuals (Ehbrecht et al. 2021). The location map of the study area and the Bayesian spatial predictions were generated using ArcMap 10.8. All other figures were plotted using R version 4.4, with the following packages: ‘brms’, ‘gghalves’, ‘tidybayes’, ‘tidyverse’, ‘Matrix’, and ‘ggplot2’.

Bayesian models were fitted using the brms package in R, which performs Bayesian inference via Stan. Weakly informative normal priors were assigned to all fixed-effect coefficients and to the standard deviations of random effects. These priors provide mild regularization while allowing the data to dominate posterior inference. In the Bayesian framework, prior distributions were combined with the likelihood function to obtain posterior distributions of model parameters. Posterior sampling was conducted using the Hamiltonian Monte Carlo (HMC) algorithm with the No-U-Turn Sampler, which efficiently explores posterior distributions and typically results in high acceptance probabilities. For each model, posterior parameter estimates were derived from four independent chains of 12 000 samples each, discarding the initial 6000 iterations as burn-in. To prevent divergence during the post-warm-up transformation, the adapt-delta parameter was adjusted to achieve an average acceptance probability of 0.99 (adapt_delta = 0.99). Because HMC sampling produces weakly autocorrelated posterior samples, chain thinning was not applied. Convergence of the Markov chains was assessed using posterior sample trace plots and the Gelman–Rubin convergence statistic (Rhat < 1.02; Gelman and Hill 2006). Additionally, we present the correlation matrix of the posterior distributions of the parameters (Supplementary Fig. S3).

## Results

### Bayesian spatial prediction of soil organic carbon distribution and storage

**Figure 2:**
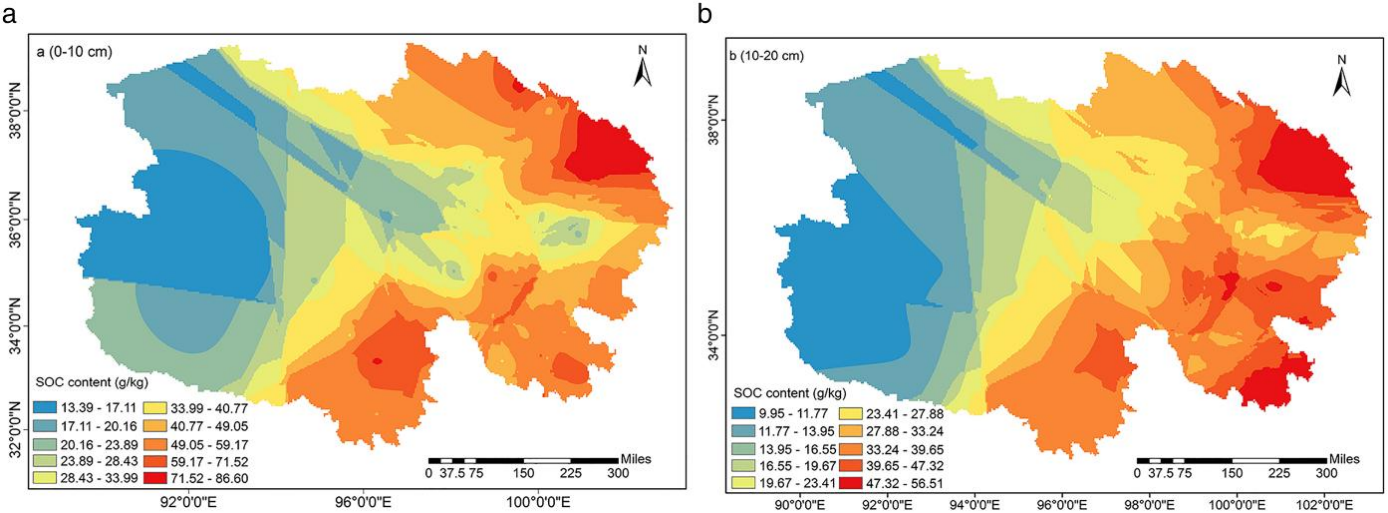
Predicted spatial distribution of soil organic carbon content in the northeastern Qinghai-Tibet Plateau at the 0–10 and 10–20 cm soil depths.

The posterior mean SOC storage was 0.37 Pg C (95% credible interval: 0.34–0.41 Pg) for the 0–20 cm profile, indicating that the study area possesses a significant carbon storage capacity (Supplementary Fig. S1). The residuals showed no evident trend or heteroscedasticity across predicted SOC values, indicating an adequate model fit. Subsequently, we conducted spatial interpolation of SOC content for the two soil profiles using empirical Bayesian spatial prediction to further explore the spatial distribution of SOC in the northeastern Qinghai-Tibet Plateau (Fig. 2). The results showed that SOC content in both the 0–10 and 10–20 cm layers exhibited a gradually decreasing trend from east to west. With increasing soil depth, the high-value areas of SOC content showed substantial differences (0–10 cm: 71.52–86.60 vs. 10–20 cm: 47.32–56.51). In contrast, the low-value areas exhibited relatively minor changes (0–10 cm: 13.39–17.11 vs. 10–20 cm: 9.95–11.77).

### Vertical distribution characteristics of soil organic carbon

**Figure 3:**
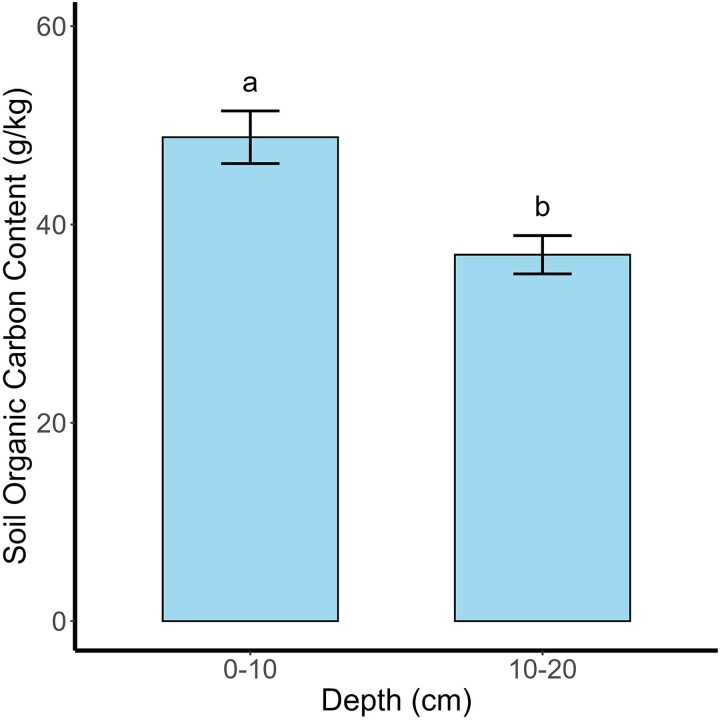
Mean soil organic carbon content at the 0–10 cm and 10–20 cm soil depths. Error bars represent standard error, and lowercase letters indicate significant differences.

SOC content varied between 8.02 and 113.11 g/kg in the surface layer (0–10 cm) and ranged from 4.28 to 93.44 g/kg in the subsurface (10–20 cm) layer. The vertical distribution of SOC content was as follows: 0–10 cm (mean: 48.80 ± 1.66 g/kg) > 10–20 cm (mean: 36.95 ± 1.93 g/kg; Fig. 3). SOC content declined markedly with soil depth, exhibiting a reduction of 24.28%.

### The influence of environmental factors on soil organic carbon

**Figure 4:**
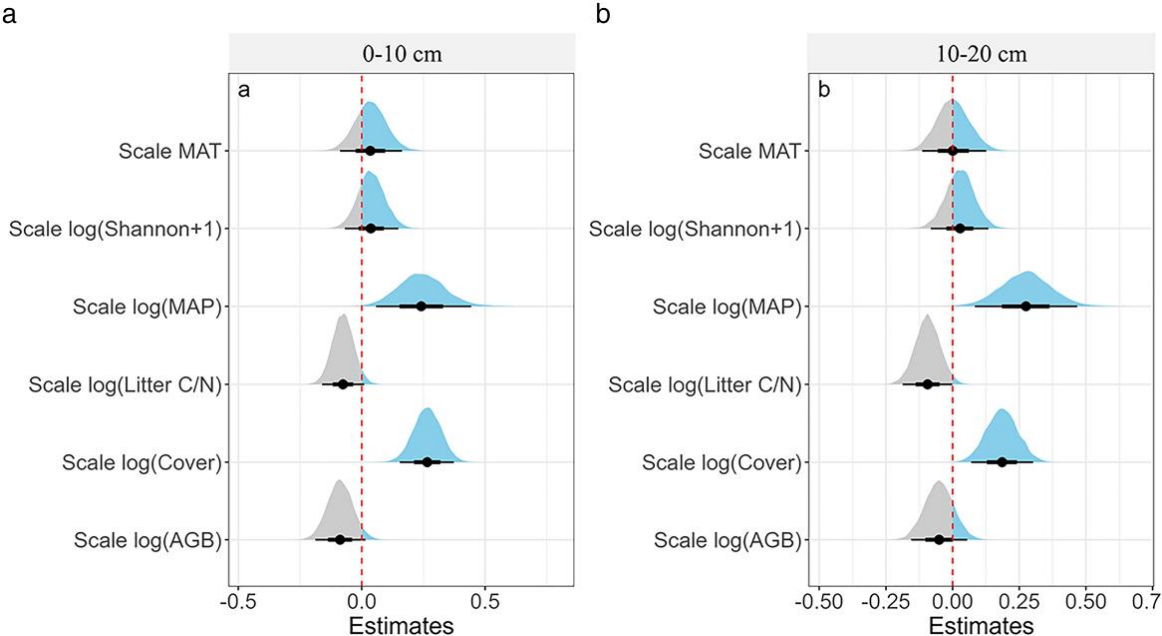
Effects of log-transformed and standardized regression coefficients for the effects of environmental factors {Shannon–Wiener diversity [Scale log(Shannon + 1)], plant cover [Scale log(Cover)], MAT (Scale MAT), MAP [Scale log(MAP)], Litter *C*/*N* ratio [Scale log(Litter *C*/*N*)], and above-ground biomass [Scale log(AGB)]} on soil organic carbon at 0–10 cm (a) and 10–20 cm (b) soil depths. It shows the parameter distribution along with the mean values, 50%, and 95% credible intervals. The 95% credible interval does not cross zero; the effect is considered significant.

The Bayesian mixed-effects model revealed that, in the surface soil layer (*R*^2^ = 0.84, 95% credible interval, 0.647–0.995), the relative impact of environmental factors on SOC was ranked as follows: plant cover (0.27), MAP (0.25), above-ground biomass (−0.09), litter *C*/*N* ratio (−0.08), Shannon–Wiener diversity index (0.04), and MAT (0.04). In the subsurface layer (*R*^2^ = 0.80, 95% credible interval, 0.559–0.994), the ranking was MAP (0.28), plant cover (0.19), litter *C*/*N* ratio (−0.09), above-ground biomass (−0.05), Shannon–Wiener diversity index (0.03), and MAT (0.0; Fig. 4). Additionally, MAP had a stronger effect on SOC in the subsurface layer than in the surface layer. In contrast, plant cover had a more pronounced effect on SOC in the surface layer than in the subsurface layer. Notably, plant cover and MAP exhibited significant positive effects on SOC in both layers, as their 95% credible interval did not overlap with zero, contrary to our second hypothesis. In contrast, other environmental factors did not significantly influence SOC, as their 95% credible intervals included zero. The posterior distributions of the model parameters and the HMC chains are shown in Supplementary Fig. S2, demonstrating that both the estimation precision and algorithm convergence are satisfactory.

### Mechanisms of environmental factors influencing soil organic carbon

**Figure 5:**
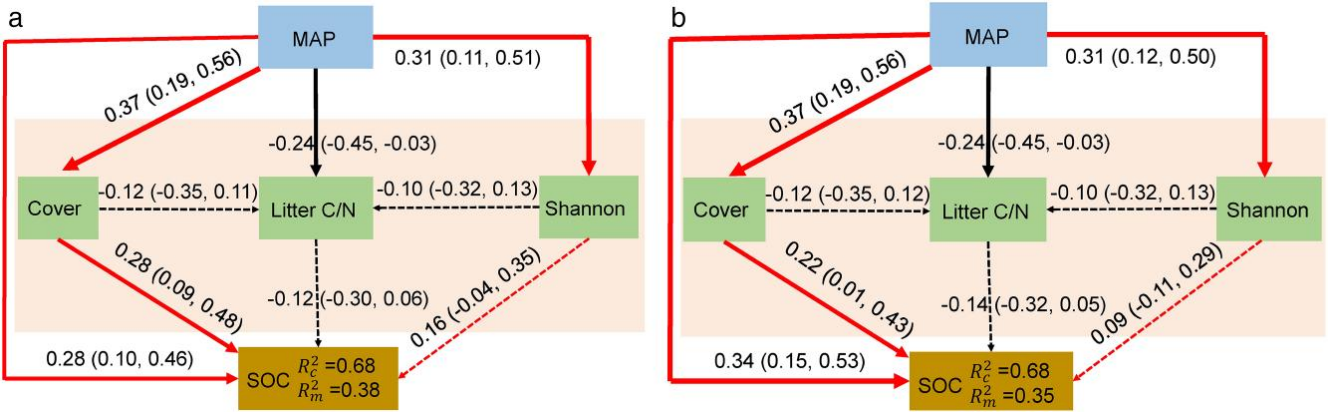
The impact of MAP, plant cover (Cover), litter *C*/*N* ratio (Litter *C*/*N*), and Shannon–Wiener diversity (Shannon) on soil organic carbon (SOC) at the 0–10 cm (a) and 10–20 cm (b) soil depths. The numbers represent path coefficients and credible intervals. Solid lines indicate significant, dashed lines indicate non-significant, and the thickness of the lines represents the magnitude of the path coefficients.

The BSEM results showed that these environmental variables collectively explained 68% of the variation in SOC in both surface and subsurface layers (Fig. 5). Among the tested variables, plant cover (path coefficient: 0–10 cm: 0.28, 10–20 cm: 0.22) and MAP (path coefficient: 0–10 cm: 0.28, 10–20 cm: 0.34) had significant positive direct effects on SOC, as their 95% credible intervals did not overlap with zero. In contrast, the Shannon–Wiener diversity index showed a non-significant positive effect (path coefficient: 0–10 cm: 0.16, 10–20 cm: 0.09), and the litter *C*/*N* ratio exhibited a non-significant negative effect (path coefficient: 0–10 cm: −0.12, 10–20 cm: −0.14), with their 95% credible intervals including zero. In addition to their direct effects, MAP also indirectly influenced SOC accumulation in both surface and subsurface layers through positive effects on plant cover and plant diversity and an adverse influence on the litter *C*/*N* ratio. Similarly, both plant cover and diversity indirectly affected SOC via their influence on litter quality (litter *C*/*N* ratio). These results highlight the combined roles of direct climatic effects and biotic mediation pathways in shaping SOC dynamics across the two soil depths.

**Figure 6:**
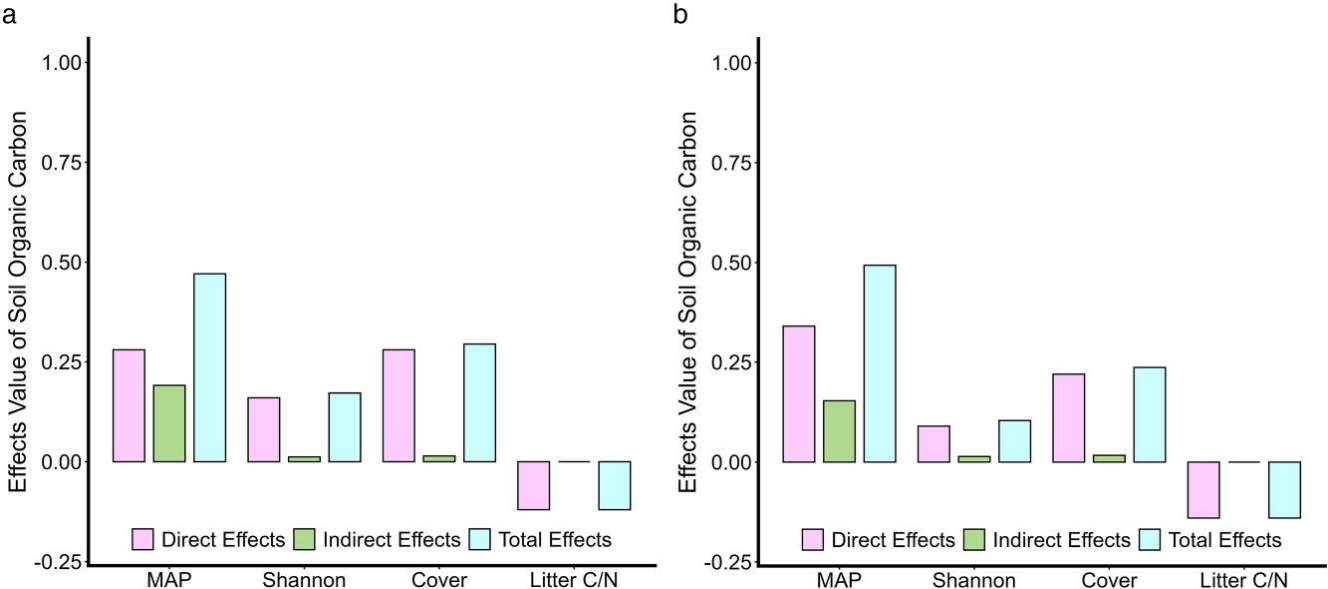
Direct, indirect, and total effects of MAP, plant cover (Cover), litter *C*/*N* ratio (Litter *C*/*N*), and Shannon–Wiener diversity (Shannon) on soil organic carbon (SOC) in 0–10 cm (a) and 10–20 cm (b) soil depths.

Overall, among the environmental variables assessed, MAP exhibited the strongest total effect on SOC accumulation in both layers, followed by the plant cover (Fig. 6). The total effect of vegetation cover on SOC was substantially greater than that of the Shannon–Wiener diversity index, underscoring the dominant role of plant cover over diversity in regulating SOC accumulation in the topsoil. For all four variables—MAP, plant cover, Shannon–Wiener diversity, and litter *C*/*N* ratio—their direct effects on SOC were notably stronger than their indirect effects. Among these, the litter *C*/*N* ratio exerted a negative total effect on SOC, whereas MAP, plant cover, and diversity had positive total effects.

## Discussion

SOC on the Qinghai-Tibet Plateau is jointly regulated by environmental factors such as climatic variables and vegetation characteristics, leading to significant regional differences in its spatial distribution. Understanding the driving mechanisms behind these regional differences can effectively enhance carbon sink capacity. Previous studies have mostly focused on the effects of plant community composition on SOC, while generally neglecting the role of plant spatial structural attributes. As a core attribute of plant spatial structure, plant cover plays a key ecological role in SOC accumulation and the formation of its spatial differentiation patterns by regulating litter input and surface physical protection. In view of this, this study employed a Bayesian model incorporating key factors including plant cover and climatic variables to systematically investigate the driving mechanisms of multiple environmental factors on SOC, confirming the important regulatory roles of precipitation and plant cover in the process of SOC accumulation in the northeastern Qinghai-Tibet Plateau.

### Spatial variation of soil organic carbon

In this study, SOC exhibited an overall decreasing trend from east to west, which is broadly consistent with machine learning predictions of soil organic carbon spatial distribution on the Qinghai-Tibet Plateau (Mao et al. 2026). From the perspective of vegetation types within the study area (Fig. 1), the eastern region is characterized by higher vegetation cover, dominated by grasslands and often accompanied by shrub and forest ecosystems. In contrast, the western region frequently experiences extreme climatic conditions, with low temperatures and limited precipitation (Li et al. 2017), resulting in sparse vegetation cover and a landscape predominantly covered by desert, which constrains the accumulation and preservation of soil organic carbon (Davidson and Janssens 2006). The unique alpine climatic pattern and zonal distribution of vegetation communities across the Qinghai-Tibet Plateau jointly regulate the accumulation and decomposition processes of soil organic carbon, thereby resulting in significant spatial differentiation and discontinuous distribution patterns of soil organic carbon at the regional scale (Fig. 2).

Additionally, SOC content was notably greater in the surface layer (48.80 g/kg) compared with the subsurface layer (36.95 g/kg), reflecting a decrease with increasing soil depth. This pattern aligns with results from a global-scale study across forest and grassland ecosystems (Takele et al. 2025). While the depth distribution of SOC exhibits a similar declining trend in both forest and grassland ecosystems, the underlying mechanisms are governed by distinct drivers. This is primarily attributed to fundamental differences between the two vegetation types in their vertical root distribution patterns and aboveground and belowground biomass allocation strategies. Root distribution determines the direct input position of carbon within the soil profile, while biomass allocation regulates the amount of litter carbon reaching the soil surface (Jobbágy and Jackson 2000). Grassland root systems are shallower than those of forests but still contribute continuously in deeper soil layers, whereas forest roots, despite penetrating deeper, exhibit higher concentrations in the surface layer (Jackson et al. 1996). Furthermore, grasslands have a higher root-to-shoot ratio reaching 3–4 in temperate grasslands compared with ∼0.26 in temperate forests (Jackson et al. 1996). This indicates that grassland ecosystems allocate more photosynthetically fixed carbon belowground, with SOC accumulation relying primarily on root turnover and rhizodeposition; in contrast, forests allocate more carbon above ground, with SOC accumulation depending mainly on surface litter input. In summary, as soil depth increases, the diminished inputs of surface-derived plant residues (Li et al. 2019) coupled with reduced root biomass (Frank 2002) progressively limit the available organic substrates. Consequently, the SOC content decreases in deeper soil layers.

### Climate effects on soil organic carbon accumulation

We employed Bayesian analysis to assess the environmental drivers and depth-dependent patterns of SOC accumulation in the northeastern Qinghai-Tibet Plateau. Our findings revealed that MAP exerted a significant positive effect on SOC accumulation, whereas MAT showed a positive but non-significant trend. Therefore, we conclude that water availability, rather than temperature, serves as the more critical limiting factor in the northeastern Qinghai-Tibet Plateau. This suggests that the ecological processes governing SOC accumulation are dominated by moisture conditions, thereby masking the effect of MAT. The observed MAP range (209.80–792.40 mm) reflects substantial spatial heterogeneity in moisture availability, supporting the broader applicability of our results. Increased rainfall enhances soil moisture. Soil moisture is a key factor that limits vegetation growth on the Qinghai-Tibet Plateau (Yang et al. 2013). Higher soil moisture content reduces the microbial decomposition of SOC (Chen et al. 2020), which, in turn, leads to an increase in SOC accumulation.

Previous studies have indicated that temperature exerts a greater influence on SOC than precipitation (Wang et al. 2004, Allen et al. 2013); however, the results of this study contradict these findings. Recent studies on climatic factors suggest that, compared with temperature, hydrological conditions exert a greater influence on carbon storage (Mishra et al. 2021, Liu et al. 2024) in alpine grasslands. This discrepancy may be due to the water limitations that vegetation faces on the Qinghai-Tibet Plateau. Rainfall, as a crucial alternative water source, stimulates grassland productivity (Wang et al. 2024) and significantly increases plant cover (Supplementary Fig. S4), thus enhancing the organic carbon input into the soil (Tang et al. 2018, Bai and Cotrufo 2022).

### The role of vegetation cover in driving soil organic carbon accumulation

The association of vegetation attributes with SOC has been a hot topic of ecological research, particularly in elucidating the mechanisms driving carbon sequestration. Vegetation cover is a primary source of SOC. Nevertheless, existing research on vegetation community structural attributes affecting SOC has predominantly emphasized community species diversity and biomass (Chen et al. 2018, Spohn et al. 2023), often overlooking the role of plant cover in SOC. Earlier research has also shown that plant diversity significantly influences SOC (Lange et al. 2015). In contrast, our results demonstrated that plant diversity had no significant effect on SOC, whereas plant cover exerted a significant positive influence, which aligns with a recent synthesis across tropical savannas (Zhou et al. 2023). Plant cover directly influenced both the above-ground (Li et al. 2026) and belowground biomass (Vicente-Vicente et al. 2017) and contributed to surface litter accumulation (Kadovic et al. 2012), all of which together determine the SOC input.

Furthermore, plant cover enhances soil physical and chemical properties by providing physical protection to the soil surface (Ruiz-Colmenero et al. 2013), supplying organic matter from plant canopies and root systems (Bronick and Lal 2005), and promoting the stability of SOC structure through increased SOC accumulation and the formation of aggregates (Six et al. 1998). Additionally, plant cover influences the soil water balance by facilitating the development of large pores through biological activities and enhancing soil structural stability (Bronick and Lal 2005). In general, higher plant cover leads to reduced erosion and less SOC loss (El Kateb et al. 2013, Eshghizadeh et al. 2016). Importantly, plant cover is regulated by climatic factors such as MAP, which further links environmental conditions to SOC accumulation, a pattern supported by a long-term field experiment on precipitation variation in the northeastern Qinghai-Tibet Plateau (Deng et al. 2024). This experiment found that reduced rainfall significantly decreased plant cover and diversity, consistent with the results of our BSEM (Fig. 5). This could be attributed to lower rainfall, which reduces surface soil moisture, causing plants to experience drought stress, accelerating senescence and death, and leading to a corresponding decline in plant cover and diversity in the region. Taken together, these results indicate that the MAP shapes plant cover (and to a lesser extent, diversity) by regulating soil moisture, whereas plant cover directly and indirectly promotes SOC accumulation through multiple mechanisms. In summary, MAP and plant cover play crucial and irreplaceable roles in increasing SOC accumulation and enhancing soil carbon sequestration capacity.

### Effect of litter *C*/*N* on soil organic carbon accumulation and its depth-dependent pattern

Across the Qinghai-Tibet Plateau’s grassland ecosystems, grassland communities with high plant cover and diversity usually produce low litter *C*/*N*, which can promote SOC accumulation. This finding aligns with prior research (Zhou et al. 2019). Litter *C*/*N* is a key factor controlling the rate of litter decomposition (Adair et al. 2010). The deposition of plant litter on the soil surface marks the initiation of microbial-mediated carbon transformation from plants to soil (Liang et al. 2017). During the early stages of decomposition, a significant amount of plant-derived carbon is retained in mineral soil (Cotrufo et al. 2015). According to the microbial efficiency-matrix stabilization framework, high-quality litter (characterized by a low litter *C*/*N* ratio) typically promotes the synthesis of microbial products and necromass, thus enhancing the formation and persistence of MAOC (Kallenbach et al. 2016, Liang et al. 2019), thereby leading to an accumulation of SOC. In contrast, systems with low-quality litter (high litter *C*/*N*) exhibit a pronounced home-field advantage (HFA), whereby microorganisms are more likely to decompose litter from their own community, especially in environments dominated by low-quality litter. Consequently, a negative relationship between the litter *C*/*N* ratio and SOC content was expected. However, this study lacked microbial validation, and future research should focus on elucidating the underlying microbial mechanisms.

Notably, the Bayesian mixed-effects model revealed a depth-dependent pattern in environmental controls over SOC accumulation, with MAT and plant cover exerting stronger effects on surface soil than on the subsurface layer. Earlier research has shown that the impact of climatic factors on SOC decreases as soil depth increases (Badgery et al. 2013), a pattern that has also been observed in the Qinghai-Tibetan Plateau ecosystem (Tian et al. 2007). Compared with surface soil (0–10 cm), the buffering effect of deep soils weakens the influence of temperature on organic carbon in the subsurface layer (10–20 cm), where organic carbon accumulation is lower, thus resulting in a smaller temperature effect on the organic carbon of deeper soil layers (Zhang et al. 2022). Furthermore, the influence of plant cover on SOC diminishes with soil depth, primarily because of the reduced input of plant-derived carbon, such as litter and root exudates (Vicente-Vicente et al. 2017). Although this study elucidates the depth-dependent patterns of SOC accumulation, its scope is confined to the 0–20 cm soil layer. Future research should extend investigations to decipher SOC accumulation mechanisms in deeper soil profiles (>20 cm), where carbon dynamics are poorly quantified.

### Limitation and future research direction

A limitation of our study is that we did not measure soil moisture content across our study sites, and no high-resolution soil moisture datasets were available for inclusion in our analyses. Soil moisture is a critical mediator of climate effects on soil carbon dynamics, and future research incorporating in situ or remotely sensed soil moisture data will help to more fully disentangle the complex relationships between climate, vegetation, and SOC accumulation in this alpine grassland region.

## Conclusion

This study employed Bayesian analysis, revealing the depth distribution of SOC in the northeastern Qinghai-Tibet Plateau and clarifying the roles of precipitation and plant cover as key drivers of SOC accumulation. The results indicated a significant decrease in SOC with increasing soil depth. MAP and plant cover were the dominant factors influencing SOC accumulation in both surface and subsurface soils. The influence of temperature and plant cover on SOC diminishes with depth. This reveals a pronounced depth-dependent pattern of SOC accumulation across the Qinghai-Tibet Plateau. Consequently, future studies need to incorporate differential driver weighting—prioritizing vegetation cover and thermal regimes in the surface layer while emphasizing precipitation controls in the subsurface layer—to correct systematic estimation biases, and future ecosystem restoration initiatives should correspondingly prioritize enhancing vegetation cover.

## Supplementary Material

plag024_Supplementary_Data

## Data Availability

Raw data and R code are available online at https://doi.org/10.17632/s36jhr93gy.1.
