# Supplementary material for: Precipitation and plant cover promote soil organic carbon accumulation in the northeastern Qinghai-Tibet Plateau
Source: AoB Plants. 2026 Jun 2;18(3):plag024. doi: 10.1093/aobpla/plag024 (PMC13278778; doi:10.1093/aobpla/plag024)

Supplementary Material

Article title: **Precipitation and plant cover promote soil organic carbon accumulation in the northeastern Qinghai–Tibet Plateau**

Table S 1. Latitude, longitude, mean annual temperature (MAT), and mean annual precipitation (MAP) of the sampling sites.

Site	Grassland type	X-coord	Y-coord	MAT /℃	MAP /mm
1	Temperate steppe	35.7759	101.1698	1.18	531.30
2	Temperate steppe	35.7139	100.9311	1.33	515.10
3	Temperate steppe	35.7105	100.9358	1.33	515.10
4	Temperate steppe	35.5714	101.0748	1.38	542.30
5	Temperate steppe	32.7777	100.5641	-2.88	765.60
6	Temperate steppe	36.1059	100.1059	3.32	425.60
7	Temperate steppe	34.1615	95.8259	-1.31	547.00
8	Temperate steppe	34.1626	95.8255	-1.31	547.00
9	Temperate steppe	34.8453	94.9171	-2.60	366.90
10	Temperate steppe	34.8436	94.9164	-2.60	366.90
11	Temperate steppe	33.8008	92.2966	-3.10	352.60
12	Temperate steppe	37.2764	100.3265	0.05	520.50
13	Temperate steppe	37.1073	99.7577	0.17	440.40
14	Lowland meadow	34.6648	101.7489	0.07	664.60
15	Lowland meadow	34.3400	100.5720	-3.15	662.80
16	Lowland meadow	34.3765	100.4966	-2.38	650.80
17	Lowland meadow	34.3659	100.4938	-1.52	642.10
18	Lowland meadow	34.3583	100.4924	-1.52	643.80
19	Lowland meadow	34.4268	100.2809	-0.11	584.70
20	Lowland meadow	33.8391	99.7038	-1.17	608.60
21	Lowland meadow	33.6005	99.9009	-2.67	672.90
22	Lowland meadow	33.7060	99.4534	-2.96	613.60
23	Lowland meadow	33.7669	99.4412	-0.68	579.40
24	Lowland meadow	33.3877	100.4986	-0.76	740.20
25	Lowland meadow	33.3862	100.4935	-0.56	739.30
26	Lowland meadow	34.6351	98.0113	-3.16	395.00
27	Lowland meadow	34.6367	98.0157	-3.10	400.20
28	Alpine meadow	37.0273	98.6974	-2.11	263.70
29	Alpine meadow	35.7205	99.5439	-2.42	450.70
30	Alpine meadow	34.5265	97.9850	-4.08	496.70
31	Alpine meadow	33.8368	97.1685	-4.20	639.20
32	Alpine meadow	33.8714	97.2221	-4.85	641.60
33	Alpine meadow	32.3070	96.3043	-3.15	697.80
34	Alpine meadow	33.3080	96.3052	-2.18	632.60
35	Alpine meadow	33.5735	96.0410	-2.94	605.40

36	Alpine meadow	33.8230	95.7082	-0.50	545.90
37	Alpine meadow	34.1038	95.8123	-1.52	533.70
38	Alpine meadow	34.1299	95.8533	-1.62	549.40
39	Alpine meadow	34.1457	95.8606	-2.39	564.70
40	Alpine meadow	34.1455	95.8605	-2.39	564.70
41	Alpine meadow	36.6368	100.0139	0.12	446.70
42	Alpine meadow	37.0220	100.7527	-1.84	546.40
43	Alpine meadow	37.0223	100.7529	-1.84	546.40
44	Alpine meadow	37.9052	100.3786	-5.82	541.90
45	Alpine meadow	37.9077	100.3756	-5.82	541.90
46	Alpine meadow	38.0059	100.2574	-7.38	562.40
47	Alpine meadow	38.5954	99.3847	-5.46	455.20
48	Alpine meadow	38.4679	99.5364	-4.97	472.80
49	Alpine meadow	37.8698	101.0450	-5.19	559.70
50	Alpine meadow	37.6111	101.3133	-2.23	554.30
51	Alpine meadow	37.6113	101.3040	-2.11	556.40
52	Alpine meadow	37.7015	101.2655	-2.65	561.20
53	Alpine meadow	37.6676	101.1770	-1.65	559.00
54	Alpine meadow	37.6693	101.1773	-1.65	559.00
55	Temperate meadow steppe	35.2981	101.2756	-0.33	596.50
56	Temperate meadow steppe	35.2924	101.2714	-0.33	596.50
57	Temperate meadow steppe	35.2835	101.3253	-0.85	603.90
58	Temperate meadow steppe	35.0825	101.4758	-1.05	634.90
59	Temperate meadow steppe	33.3197	99.1826	-7.22	657.10
60	Temperate meadow steppe	35.7701	99.8891	-0.27	462.00
61	Temperate meadow steppe	36.7542	99.7386	0.39	425.80
62	Mountain meadow	34.6647	100.8674	1.09	597.40
63	Mountain meadow	34.9721	100.8661	-1.48	605.10
64	Mountain meadow	34.9703	100.8650	-1.48	605.10
65	Mountain meadow	34.3936	100.2729	-0.51	592.90
66	Mountain meadow	34.1506	100.1599	-2.67	640.70
67	Mountain meadow	34.1555	100.1600	-2.67	640.70
68	Mountain meadow	34.3297	100.6097	-4.04	673.90
69	Mountain meadow	34.3285	100.6076	-4.04	673.90
70	Mountain meadow	34.0821	100.1051	-3.92	651.90
71	Mountain meadow	34.0209	100.0841	-4.15	655.40
72	Mountain meadow	33.9557	99.8100	-2.65	616.60
73	Mountain meadow	33.9389	99.8108	-2.64	619.30
74	Mountain meadow	33.3913	100.2020	-2.73	713.80
75	Mountain meadow	33.3912	100.9275	-3.75	782.70
76	Mountain meadow	32.7784	100.5619	-2.56	762.20
77	Mountain meadow	33.2913	100.8695	-3.14	779.20
78	Mountain meadow	33.2930	100.8669	-3.24	792.40

79	Mountain meadow	33.4403	100.0617	-2.96	701.50
80	Mountain meadow	33.2831	100.4084	-2.59	743.90
81	Mountain meadow	34.7984	98.1229	-3.74	381.50
82	Mountain steppe	35.0262	98.6297	-5.60	409.70
83	Herbaceous marsh	34.9536	98.9326	-7.07	466.90
84	Herbaceous marsh	34.2275	97.8140	-6.82	596.20
85	Herbaceous marsh	34.2285	97.8128	-6.82	596.20
86	Alpine steppe	35.1128	97.9746	-3.78	348.20
87	Alpine steppe	35.1124	97.9886	-3.80	348.50
88	Alpine steppe	35.0341	98.0724	-3.76	350.40
89	Alpine steppe	34.8667	98.2940	-3.27	375.80
90	Temperate desert steppe	35.2577	93.1281	-4.59	324.70
91	Temperate desert steppe	35.2575	93.1282	-4.59	324.70
92	Temperate desert steppe	34.2094	92.4380	-3.26	323.00
93	Temperate desert steppe	34.2092	92.4394	-3.26	323.00
94	Temperate desert steppe	33.8003	92.3000	-3.10	353.20
95	Alpine meadow steppe	37.0686	101.0660	-1.48	569.70
96	temperate desert steppe	36.9606	98.4335	3.46	209.80
97	Alpine meadow	37.6204	101.2978	-2.24	554.00
98	Mountain meadow	33.2913	100.8695	-3.14	779.20

Supplementary Figure

Fig. S.1. Posterior distribution (left) and residual distribution (right) for SOC storage estimation in Qinghai grasslands.

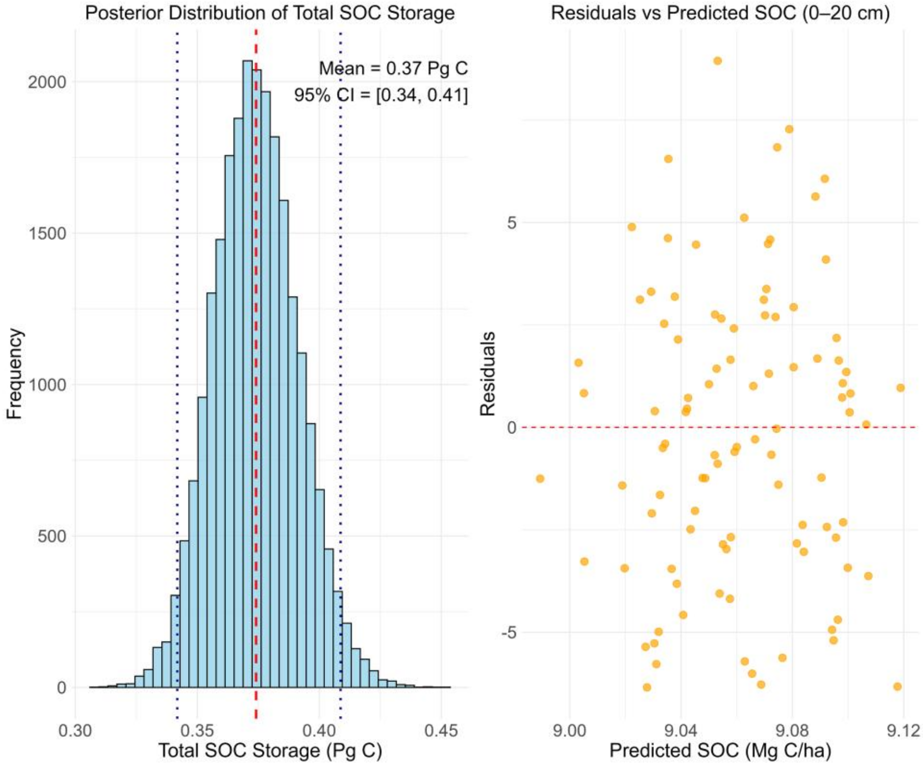

Fig. S.2. Fig. S. 2. Posterior distributions (left panels) and Hamiltonian Monte Carlo (HMC) chain convergence (right panels) of parameters (Scale log(Shannon+1), Scale log(Cover), Scale MAT, Scale log(MAP), Scale log(Litter C/N), and Scale log(AGB)) from the Bayesian mixed-effects model for the 0-10 cm (a) and 10-20 cm (b) soil depths. Each histogram (blue) represents the posterior distribution of the parameter, with density on the y-axis. The HMC chains (black lines) for the four independent chains (Chains 1-4) demonstrate convergence (stability over iterations) and mixing behavior (adequate exploration of the posterior space).

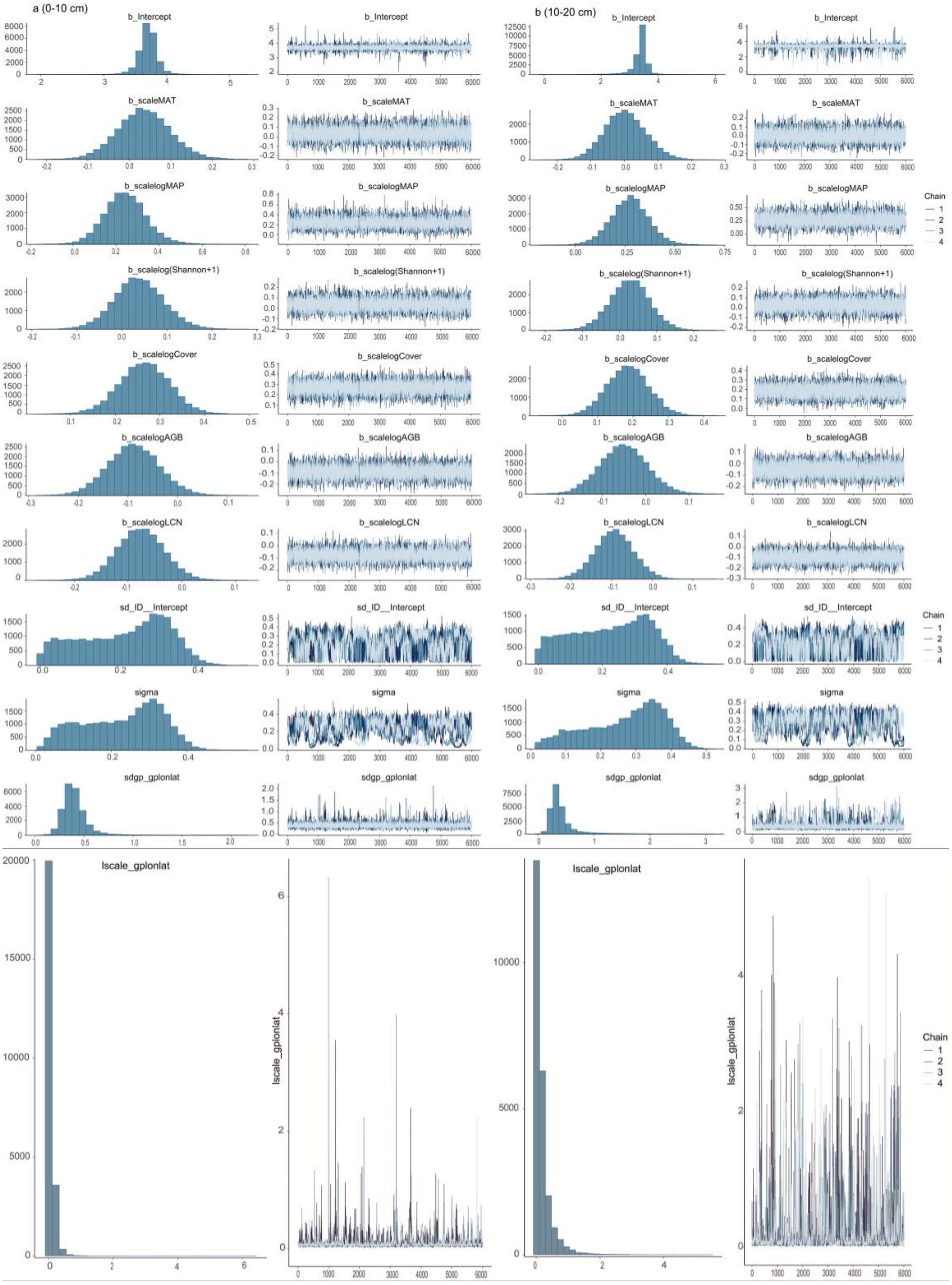

Fig. S.3. Correlation matrix of posterior parameters from the Bayesian mixed-effects model.

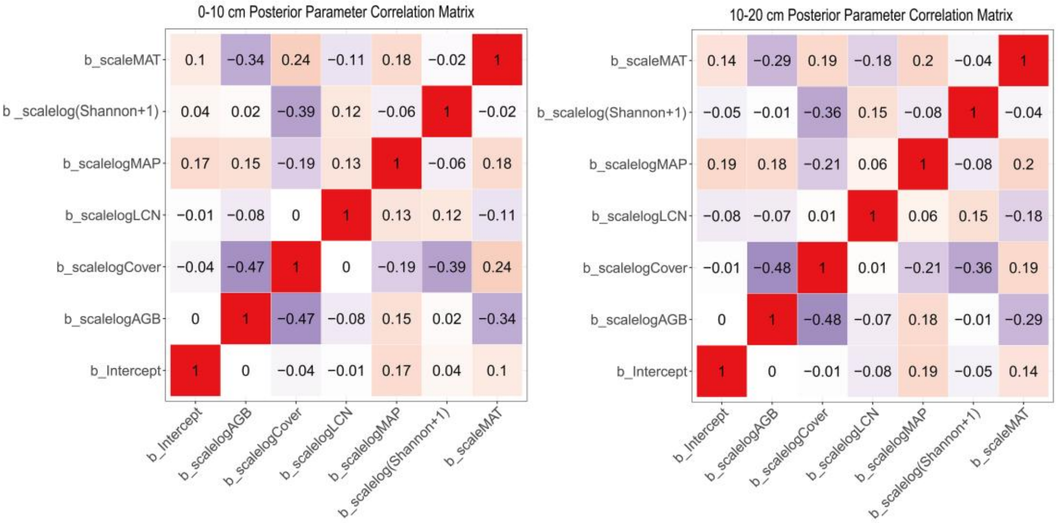

Fig. S.4. Relationship between mean annual precipitation and plant cover. The shaded area represents the 95% credible interval of the fitted line. The solid line indicates a significant relationship (the 95% credible interval does not overlap with zero).

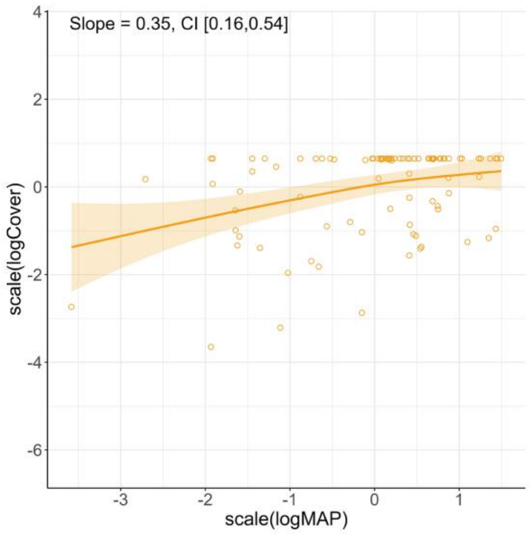

Supplement: plag024_Supplementary_Data [file plag024_supplementary_data.pdf]
